# Assessing the Impact of Home Environmental Exposures on Allergic Rhinitis Using Real-Time Air Quality Monitoring and Symptom Assessment: Observational Feasibility Study

**DOI:** 10.2196/73215

**Published:** 2025-06-23

**Authors:** Aero Cavalier, Anthony I Dick, Vickie Johnson II, Emily Cramer, Kamal Eldeirawi, Jayant Pinto, Sharmilee M Nyenhuis, Victoria S Lee

**Affiliations:** 1 Section of Allergy, Immunology, and Pulmonology Department of Pediatrics University of Chicago Chicago, IL United States; 2 Department of Otolaryngology University of Illinois Chicago Chicago, IL United States; 3 University of Illinois College of Medicine Chicago, IL United States; 4 Department of Biostatistics and Epidemiology Children's Mercy Hospital Kansas City United States; 5 Department of Population Health Nursing Science University of Illinois Chicago Chicago, IL United States; 6 Department of Otolaryngology University of Chicago Chicago United States

**Keywords:** rhinitis, allergic rhinitis, chronic rhinitis, ecological momentary assessment, EMA, air quality, home air quality, mobile phone

## Abstract

**Background:**

Rhinitis is the most common sinonasal condition and poses a significant cost burden. Rhinitis symptom control is associated with exposure to environmental triggers (eg, aeroallergens, pollutants, and irritants). While people spend much of their time at home, studies examining the association of rhinitis symptoms with home environmental exposures, especially in low-income, urban, and racial or ethnic minorities, are limited. Frequently, 3 types of surveys are used in ecological momentary assessment (EMA): a survey conducted at a predetermined rate, an event-triggered survey, and a follow-up survey to gauge behavioral changes in response to the event.

**Objective:**

This study aims to determine the feasibility and usability of daily and triggered EMA paired with an indoor air quality monitor to collect exposure and rhinitis symptom data.

**Methods:**

Participants were recruited from the Allergy and Ear, Nose, and Throat clinics at 2 academic centers. Participants had to have a rhinitis diagnosis with active symptoms, be 18 years of age or older, self-identify as a racial or ethnic minority, live in the city of Chicago, be able to read and speak English, and have a smartphone. Participants received the Awair Omni air quality monitor to measure volatile organic compounds, particulate matter, and humidity. EMA data were collected using a personal smartphone using the PiLR Health app. Participants were sent daily scheduled surveys, random check-in surveys, and air quality event-triggered survey EMA notifications to assess rhinitis symptoms, environmental exposures, and mitigation strategies for 14 days. After the 14-day data collection period, participants completed the acceptability, appropriateness, and feasibility survey items. Feasibility metrics captured included recruitment and retention, demographics, rhinitis symptoms, and the usability of the PiLR Health App and Awair Omni. Barriers and challenges were identified and captured by the study staff. Descriptive statistics were performed using Excel (Microsoft Corp).

**Results:**

A total of 24 participants were approached, 15 participants consented and 12 participants completed the study. Participants received an average of 62.42 (SD 14.26) total surveys during their study period, and of those surveys, an average of 36.83 (SD 22.18; 59%) surveys were completed. All 12 participants met the threshold for successful home air monitoring (11 days of continuous environmental data assessment). The usability of study components and integration into the overall study was high (usability scale≥68), indicating participants considered each of the devices to be usable. Participant feedback on the study was positive; yet, they did identify areas for improvement including getting air quality data in real time, providing more detailed instructions for device setup, and doing more check-ins.

**Conclusions:**

A real-time assessment of home environmental exposures and subjective rhinitis symptoms was feasible to conduct. This study will support the development of targeted interventions to address disparities in sinonasal disease care and outcomes.

## Introduction

### Background

Allergic rhinitis (AR) is the most common allergic airway disease and is characterized by nasal congestion, runny nose, sneezing, and a reduced sense of smell in response to environmental allergen triggers [1]. The prevalence of this disorder has been rising globally, reaching a range of 12%-20% in high-income countries [2]. About 70% of pharmacy customers purchasing nasal treatments were self-medicating for AR without a diagnosis, and only 44.3% of those with AR symptoms had received a clinical diagnosis [3].

AR has a significant impact on quality of life and is associated with other comorbidities such as asthma, cognitive dysfunction, and depression [4]. Economically, AR poses a significant burden on individuals, health care systems, and society. The direct medical expenditure for AR was estimated to be over US $3.4 billion, mostly from clinic visits and prescription medications [5]. AR incurred the highest indirect costs due to absenteeism or the inability of the individual to function at full capacity in the workplace, with an annual average loss of US $593 per employee, surpassing conditions such as arthritis, migraine, diabetes, and depression [1].

AR is triggered by a range of allergens, most commonly pollen (primarily outdoor), mammal or arthropod-derived allergens, and environmental pollutants, which can be indoor or outdoor [6]. Several studies have shown that indoor air quality (IAQ) in the home is directly associated with the development and severity of AR symptoms [7,8]. The IAQ is determined by factors such as outdoor air infiltration, ventilation systems, and the amount of pollutants generated indoors [9].

The most common indoor pollutants that trigger AR include volatile organic compounds (VOCs), particulate matter (PM), passive tobacco smoke, carbon monoxide, formaldehyde, and greenhouse gases like ozone, sulfur dioxide, and nitrous oxide [10]. These are mostly generated from daily household activities including sanitizing, heating, cooking, burning candles, and smoking [11-13].

The US Environmental Protection Agency estimates that US residents aged 18-65 years spend an average of 80% of the day indoors. A large portion of this indoor time is spent at home [14]. To develop targeted strategies and therapeutic measures to control these environmental factors, it will be vital to analyze them, and how they influence the development and exacerbation of AR.

The predominant methods for the assessment of the relationship between home IAQ data and sinonasal symptoms include periodic patient self-reports on home characteristics and the collection of samples from homes. These are subjective, prone to recall bias, and often capital-intensive [15]. More so, they have no room for real-time evaluation of adjustable home environmental factors and provision of interventions that may reduce exposure to triggers.

Ecological momentary assessment (EMA), also called experience sampling, is a data collection technique where people provide real-time reports on their experience, including their symptoms, actions, and environment [16]. This method usually involves multiple, frequent measurements across a period of time, offering an opportunity for real-time collection of longitudinal data [16]. EMA can inform understanding of the real-time relationship between the environment and symptoms of sinonasal disease. EMA initially used handwritten logs but has since evolved with technology to use automated logs in mobile devices leading to an increased use of EMA in the study of diseases affected by environmental exposures [17,18]. Our prior study, however, showed that its adoption in rhinology studies remains limited [19]. Furthermore, the advent of commercial air monitoring devices, such as the Awair system, presents an opportunity to supplement the EMA symptoms data with continuous home IAQ data [20]. Despite the availability of these tools for real-time assessment of home environmental exposure, no studies have applied them to assess the relationship between home environmental exposures and AR symptoms.

### Objectives and Aims

This study aimed to determine the feasibility and usability of (1) EMA to assess self-reported home environmental exposures (eg, aeroallergens or irritants) and rhinitis symptoms (sneezing, itching, and nasal congestion or drainage), and (2) real-time objective measures of home environmental exposures (VOCs, PM_2.5_ [particulate matter with a diameter of ≤2.5 µm], and humidity) using a home air quality monitor (Awair) over a 2-week study period. We assessed the feasibility of using the Awair Omni monitors to collect measures of IAQ such as humidity, VOCs, and PM_2.5_ in real time. This multifaceted approach enables us to notify participants of elevated indoor environmental pollutants while simultaneously gathering real-time data on IAQ and AR symptoms. This paper outlines the feasibility and usability results of this study.

## Methods

### Ethical Considerations

All studies were conducted in accordance with the Declaration of Helsinki and the International Conference on the Harmonization of Good Clinical Practice guidelines and approved by the relevant institutional review boards at the University of Chicago (IRB #22-1469) and the University of Illinois Chicago (IRB #2022-0361). All participants were required to provide written consent to participate prior to any study procedures. All study data were deidentified before being shared. Participants were given a study ID and only site-specific research teams had access to those codes. Participants who completed all study activities and returned the device were given US $60 via physical or electronic gift card.

### Study Components

The Awair Omni IAQ Monitor continuously monitors about 1000 square feet of indoor air for 7 air quality indicators: total VOC (TVOC), PM_2.5_, temperature, humidity, carbon dioxide, ambient light, and ambient noise. This study only focused on humidity, VOCs, and PM_2.5_ levels because of their known associations with rhinitis symptoms. The detectable ranges for the Awair Omni were 0-60,000 ppb/±10% for TVOCs, 0-1000 μg/m^3^/±15 μg/m^3^ or 15% for PM_2.5_, and 0% to 100%/±2% RH for humidity. The acceptable ranges were <333 ppb for TVOCs, <15 μg/m^3^ for PM_2.5_, and >30% to <60% for humidity. The Awair Omni device measures about 4×4×1.3 inches, is Wi-Fi– and Bluetooth-enabled, plugs into a standard alternating current outlet, and includes an 8-hour battery backup. Participants were asked to place their device in either their bedroom or living room in their home. Participants who did not have access to stable Wi-Fi in their homes were ineligible to participate. An air quality reading was taken every 10 seconds and real-time data were uploaded to a dashboard accessed only by research personnel. The Awair Omni proprietary dashboard provided secure communication between the Awair Omni and its dashboard. Data were exported from the dashboard as a .csv file.

EMA data were collected electronically using the PiLR EMA Health software platform, which was installed on the participant’s personal smartphone (iOS or Android). Data captured were transmitted to the PiLR EMA platform and stored in a secure database. Participants received three scheduled surveys on their smartphones each day. The first scheduled survey was the Daily Rhinitis Survey that was sent once daily at 9 AM. The next scheduled survey was the Rhinitis Check-In Survey, which was sent two times per day, once in the midlate morning and once in the afternoon. In addition to the scheduled surveys, participants received at least 2 triggered surveys when the air quality monitor values would go outside of acceptable thresholds. The first was the Triggered Air Quality Survey that was sent at the time of the event, and the second survey was the air quality follow-up survey that was sent 45 minutes after the event. The surveys were available for a limited period of time, with the Daily Rhinitis Survey available for 120 minutes, the random Rhinitis Check-In Surveys available for 60 minutes, the initial Triggered Air Quality Survey available for 45 minutes, and the air quality follow-up survey for 60 minutes (Table 1). To reduce the burden of surveys sent, all components of air quality had to return to the recommended thresholds before another air quality survey was triggered. Surveys were also disabled between 10 PM and 5 AM.

**Table 1:** The types of ecological momentary assessment surveys sent to study participants to assess rhinitis symptoms.

Survey title	Per day	Duration of availability
Daily Rhinitis Survey	1	120 minutes
Rhinitis Check-In Survey	2	60 minutes each
Triggered Air Quality Survey	Per event	45 minutes each
Follow-Up Air Quality Survey	Per event	60 minutes each

Researchers were able to access the PiLR dashboard to monitor participants’ use of the EMA app and review their data. Data were exported from the dashboard as a .csv file.

After the 14-day study period, participants were asked to complete an adapted System Usability Scale (SUS) on the Awair Omni, the PiLR EMA Health app, and integration of the 2 systems. Due to an error, the usability of the PiLR EMA Health app and integration of the 2 systems were collected in only half of the participants. The SUS scores were recoded and then analyzed by calculating the mean scores for the individual scales where the scheme assumed 1=strongly agree and 5=strongly disagree. These scores were then corrected so the highest potential score would be 100 and the lowest potential score would be 0.

### Participants

Participants were recruited from a convenience sample of patients attending a clinician encounter in the Allergy or Ear, Nose, and Throat (ENT) clinics at the University of Chicago Medical Center or the University of Illinois Hospital and Health System. Participants had to have a diagnosis of rhinitis with active symptoms (sneezing, itching, nasal congestion, and nasal drainage), be 18 years of age or older, self-identified as being from racial or ethnic minority groups (Hispanic, Black, American Indian, Alaskan Native, Asian, Pacific Islander, Native Hawaiian, Arab, or mixed race), live in the city limits of Chicago, be able to read and speak English, and have a smartphone. Participants who had comorbid sinonasal conditions, a sinus tumor, or a terminal illness or immune dysfunction were excluded.

### Recruitment

Potential participants were prescreened by looking through electronic health records and looking for age, race, diagnosis of rhinitis, and no history of comorbid sinonasal conditions. Study staff approached a patient, after receiving approval from the clinician, and gave a brief overview of the study. Patients who expressed interest completed a REDCap (Research Electronic Data Capture; Vanderbilt University) survey that collected current contact information and verified eligibility. In the eligibility screener, patients had to confirm that they had their own stable Wi-Fi signal that they could use 24/7 in their homes. Patients were informed that they would receive an electronic gift card and information about their air quality upon completion of the study. Eligible patients were consented onsite after completing the eligibility survey.

### Feasibility Measures

To assess the feasibility of this study, we collected measures related to recruitment and retention (Textbox 1). Recruitment and retention were determined by the number of participants recruited and the percentage of participants who completed the study after consenting, respectively. Sociodemographic information, including race and ethnicity, education level, occupation, yearly household income, and health insurance, was collected at baseline using a REDCap form that the participant completed after consent was completed. A 6-item survey called the Rhinitis Control Assessment Test and a 22-item survey called the Sino-Nasal Outcome Test were collected to evaluate different aspects of rhinitis control [21]. These were completed at baseline and the end of the study period in REDCap.

**Feasibility measures assessed in Home Air and Rhinitis study.**

**Secondary outcomes**

- Age, race, sex, primary language, household income (REDCap [Research Electronic Data Capture])
- Rhinitis control assessment and sinonasal outcome test (baseline, day 14; REDCap)

**Recruitment rate and eligibility**

- Number of people approached, screened, eligible, and ineligible (REDCap)

**Participant retention**

- Number of participants recruited (REDCap)
- Number of participants who completed 14 days of data collection (REDCap)

**Fidelity**

- Mean (SD) number of ecological momentary assessment (EMA) surveys sent and completed (PiLR Health Dashboard)
- Mean (SD) number of Awair Omni days with data (Awair Dashboard)

**Overall usability**

- Day 14 Awair Omni System Usability Scale (SUS; REDCap)
- Day 14 EMA SUS (REDCap)
- Day 14 platform integration (REDCap)

**Overall acceptability**

- Day 14 interview data

**Challenges**

- Day 14 interview, study staff notes (REDCap)

### Fidelity

Reports were downloaded from the PiLR EMA Health app to determine how many surveys were sent to participants and how many surveys were completed. Additionally, reports were downloaded from the Awair dashboard to assess the number of days the devices were online.

### Usability Assessment

After the 14-day data collection period, participants were asked to complete a short REDCap survey assessing usability (SUS) [22]. The SUS asked about participants’ experience with the Awair Omni IAQ monitor (10 items), the PiLR EMA Health app the Awair, and the integration of the two types of technology (see SUS in Multimedia Appendix 1). The responses ranged from 1=strongly agree to 5=strongly disagree. After each section, there was an open-ended item for participants to comment on what they liked or did not like about both study tools.

### Acceptability Assessment

Adherence to the EMA prompts was measured by the frequency of responses to EMA prompts over the entire study period. This includes the percentage of complete and incomplete responses to prompts out of the total number of prompts. Adherence to the Awair Omni air monitor was considered successful if participants achieved a minimum of 11 days of continued environment data assessment through Awair by keeping the device on and connected to Wi-Fi.

At the end of the study period, study staff scheduled a time to contact participants via phone or Zoom (Zoom Video Communications) to discuss their experience with the study and go over the close-out procedures. Participants were asked open-ended questions about what they liked about the study, what they disliked about the study, and suggestions for future studies. The study staff member would probe if the participant did not answer a question.

### Barriers and Challenges

During follow-up calls between participants and study staff, participants were asked questions about difficulties they have been experiencing with the air monitor or the PiLR EMA Health app. These difficulties were documented by study staff and acknowledged or addressed during larger team meetings. Study staff also encountered difficulties when setting up the device or app or while attempting to collect data. These were also documented.

## Results

### Participant Characteristics

Participants were on average 37.2 (SD 10.7) years of age (range 20-57 years) and predominantly female (n=8, 67%; Table 2). Participants self-identified as Black (n=7, 47%), Hispanic White (n=3, 20%), Asian (n=2, 13%), Native American (n=1, 7%), or belonging to more than one race (n=2, 13%). At baseline, almost all participants reported uncontrolled rhinitis symptoms (n=14, 92%) or severe sinonasal disease (n=14, 92%) based on their Rhinitis Control Assessment Test (<21) and Sino-Nasal Outcome Test scores (), respectively.

**Table 2:** Participant characteristics.

Characteristics		Value
Age (years), mean (SD)		37.2 (10.7)
Sex (female), n (%)		8 (67)
**Race and ethnicity, n (%)**		
	American Indian or Alaskan Native	1 (7)
	Asian	2 (13)
	Black or African American	7 (47)
	Hispanic White	3 (20)
	More than one race	2 (13)
**Language more comfortable speaking, n (%)**		
	English	14 (93)
	Spanish	1 (7)
**Yearly household income (US $), n (%)**		
	<30,000/year	3 (20)
	30,000-90,000/year	4 (26)
	>90,000/year	3 (20)
	Did not answer	5 (33)
RCAT^a^ score, mean (SD)		16.6 (4.7)
SNOT^b^ score, mean (SD)		51.8 (21.6)

^a^Rhinitis Control Assessment Test.

^b^Sino-Nasal Outcome Test.

### Participant Recruitment and Retention

**Figure 1:**
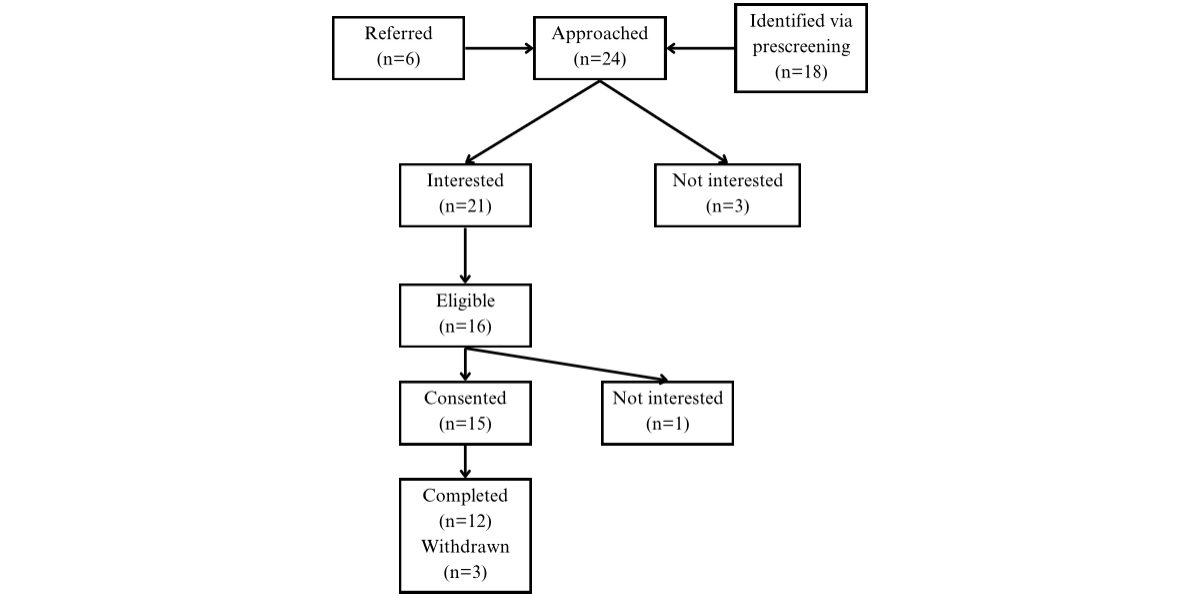
Recruitment (CONSORT) diagram for home air and rhinitis study. CONSORT: Consolidated Standards of Reporting Trials.

Figure 1 diagrams the study participant flow. A total of 24 patients were approached in the Allergy and ENT clinics, 18 patients were identified through prescreening of clinic schedules and 6 patients were identified through referral from one of the clinic physicians. Of the 24 patients approached, 21 patients expressed interest and were screened for eligibility by study staff. In total, 16 of the screened patients were eligible for the study yet 1 patient declined to consent due to being too busy to participate. A total of 15 participants consented, 3 participants withdrew from one site (reasons not provided), and 12 participants (6 from each site) completed the study.

### Fidelity

A total of 749 EMA surveys were sent to all participants for the duration of the study (Table 3). Individually, participants received an average of 62.42 (SD 14.26) total surveys during their study period, approximately 4.0 (SD 0.8) per day, and of those surveys, an average of 36.83 (SD 22.18; 59%) surveys were completed over the study period. Daily rhinitis (25.1%) and random rhinitis check-in (50.9%) surveys accounted for the majority of surveys sent to the participants. The triggered air quality and air quality follow-up surveys accounted for 11.9% and 12.2% of surveys sent, respectively. The air quality follow-up survey was the most likely to be completed (67%). Two participants did not receive any air quality surveys due to user issues with the Awair Omni air monitor. These issues included turning the Awair Omni device off or removing the device from Wi-Fi. One participant received surveys but did not complete any surveys.

**Table 3:** Number of ecological momentary assessment surveys sent to study participants.

	Daily Rhinitis Survey	Random Rhinitis Check-In Survey	Triggered Air Quality Survey	Air Quality Follow-Up Survey	All Surveys
Total surveys, n (%)	188 (25.1)	381 (50.9)	89 (11.9)	91 (12.1)	749 (100)
Number of surveys sent per participant, mean (SD)	15 (3)	31 (6)	7 (6)	7 (6)	62 (14)
Number of surveys started per participant, mean (SD)	11 (5)	18 (10)	5 (6)	6 (6)	42 (20)
Percentage of surveys started per participant (%), mean (SD)	67 (25)	56 (25)	66 (34)	74 (35)	66 (20)
Number of surveys completed per participant, mean (SD)	9 (6)	17 (12)	5 (5)	5 (5)	37 (22)
Percentage of surveys completed per participant (%), mean (SD)	59 (28)	52 (30)	53 (33)	62 (39)	58 (26)

All 12 participants met our threshold for successful home air monitoring, predetermined as a minimum of 11 days of continuous environmental data assessment. In total, 11 (92%) participants had their air monitors operational throughout the entire 14 days of their study period without significant disruption (defined as ≥2 hours of interruption) to continuous assessment of home environment data. One participant, however, had their monitor disconnected from Wi-Fi for 3 days during their study period. The quantitative data for all participants was successfully downloaded for analysis.

### Overall Usability

**Figure 2:**
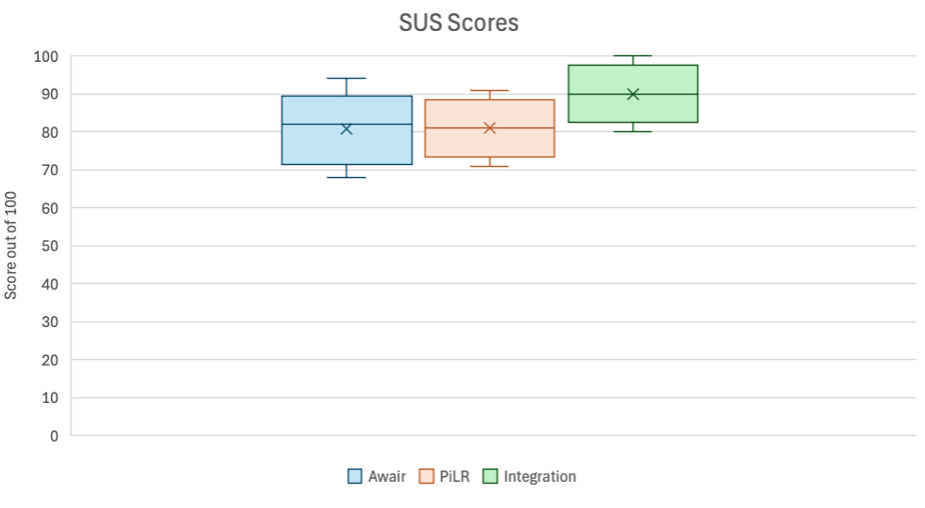
Participant reported usability of the Awair Omni air monitor and the PiLR Health app. Usability was measured by a modified SUS. SUS: System Usability Scale.

All 12 participants received and completed the SUS for the Awair. In total, 6 out of the 12 participants received and completed the SUS for the PiLR Health App and the overall integration of study components due to site coordination differences. The mean SUS score for Awair, PiLR Health App, and overall integration was high (≥68; Figure 2), indicating participants considered each of the devices to be usable.

### Overall Acceptability

Study staff notes from the 14-day interview recorded what participants liked and disliked about the study (Textbox 2). The most common things participants liked about the study was that the device was easy to use and the surveys were short and easy to complete. The most noted dislike by participants was that the PiLR Health App would send “ghost notifications” where the participant would receive a notification or sometimes the survey would not allow them to move forward, followed by participants saying the study or the instructions for setting up the air monitor were too complex for people who were less tech-savvy. Suggestions for improvement included getting their air quality data in real-time, providing video instructions for the Awair Omni device setup, and doing more frequent check-ins.

**Participant reported feasibility of study components collected via telephone at end of study.**

**Likes (n)**

- The device was easy to use (3)
- Surveys were short, easy to complete (3)
- The study was interesting (2)
- The consistency of the surveys (2)
- The device collecting air quality data (2)
- Liked the survey answers (2)
- Receiving notifications when the air quality changed (1)
- App was simple (1)

**Dislikes (n)**

- The app was “buggy” (5)
- The study or instructions were complex (4)
- None (3)
- The surveys came at an inconvenient time (3)
- Wording of questions was challenging (2)
- The Awair Omni did not have enough battery time (1)
- The device took up too much space (1)
- Surveys were too repetitive (1)
- Survey answers were not detailed enough (1)

**Suggestions for improvement (n)**

- Seeing the data in real-time or getting results faster (2)
- More frequent check-ins (2)
- Provide video instructions (1)

### Study Challenges

Study challenges were divided into the PiLR EMA Health app and the Awair Omni air monitor. The most common challenge identified was with the PiLR EMA Health App surveys. This primarily included issues with the device assignment logs, which are used to pair the PiLR EMA account to its respective Awair Omni air monitor. This resulted in a participant not receiving triggered surveys when there was a change in air quality noted by the Awair Omni. These issues were resolved by contacting the vendor and collecting information that had been overlooked previously. For the Awair Omni air monitor, the main challenge was in regard to the device shutting down temporarily or disconnecting from Wi-Fi. In one instance, a participant had changed their Wi-Fi password and had made their device disconnect from Wi-Fi. Most situations were resolved by troubleshooting with participants to determine why their devices went offline.

To address these challenges, study staff worked with vendors to troubleshoot issues, and then relayed fixes to participants. Study staff contacted and helped participants correct issues with the devices and provided information to prevent future issues. If there was a significant time of data being lost, determined by the study staff, the study period for the participant was extended to ensure that 14 days of data were collected. These barriers were recorded and modifications to our manual of operations were made to be used in future, adequately powered studies.

## Discussion

### Principal Findings

This study aimed to assess the feasibility of an air quality monitor and EMA app used simultaneously in a study exploring real-time residential Total VOC and PM_2.5_ exposures and real-time rhinitis symptoms in minoritized adults. We were able to recruit and retain participants in the study and found both the air quality monitor and EMA surveys feasible to implement with all participants keeping the air quality monitor active during the study period and approximately two-thirds of participants completing the surveys. Additionally, while we did identify some technical challenges in the pilot study, we were able to develop solutions to address the challenges and are prepared to conduct a larger, appropriately powered observational study to collect further information on rhinitis symptoms.

The field of measuring symptoms and air quality in real time in rhinitis is a nascent area, especially in minoritized populations that often have higher rates of exposure to rhinitis triggers [23]. Prior studies in real-time monitoring and rhinitis reviewed the existing research on EMA as a health monitoring tool and the use of ecological monitoring in understanding rhinitis symptoms and air quality both indoors and outdoors for individuals with asthma, but none focused on minoritized populations [19,24]. This is the only study to our knowledge that used EMA for real-time rhinitis symptoms and residential air quality monitoring in minoritized adults.

We did encounter technical challenges in regard to the PiLR EMA Health app and Awair Omni air monitor. Common challenges were participants not receiving Triggered Air Quality Surveys or the air monitor disconnecting from Wi-Fi. These challenges were technical in nature and were anticipated as this was a system never tested before in this patient population. This may have occurred at a higher rate in our participant population as Black (36%) and Hispanic (30%) households are less likely to have broadband internet than White households (21%) [25].

As research and medical care are leaning into technology more, it is important to acknowledge that there may be challenges in certain populations that could impact their ability to successfully participate in research or get medical care. Although our usability scale showed the Awair Omni air monitor and the PiLR EMA Health app to be acceptable for our participants, future research should be done that includes older participants, low-income populations, and people in digital deserts. Digital deserts are areas where there is a lack of broadband Wi-Fi, a lack of electronic devices (eg, tablets, smartphones, and personal computers), or low technological literacy. Some studies suggest that these digital deserts are primarily due to low digital literacy, language barriers, and affordability [26]. Some studies also suggest that there may be discrimination in broadband service delivery based on race and geographic location [27]. This implies that there may be a relationship between racially minoritized people having access to broadband and their ability to treat asthma symptoms. Future studies may want to consider providing participants compensation for their Wi-Fi use or providing a hotspot for the duration of their study participation.

### Limitations

Limitations to our study include a lack of broad generalizability as participants were recruited from a convenience sample of patients from Allergy and ENT clinics in urban academic centers. Further, older adults were not represented in this study sample which may have been due to the heavy technology component of our study and the requirement for Wi-Fi and a smartphone. Future studies should offer Wi-Fi or smartphones for those who do not have access to these and provide additional technology training to those requesting assistance.

### Conclusions

This was the first study to use EMA and air quality monitors to monitor rhinitis symptoms in an urban and racially minoritized population. The findings from this study will provide insight into developing research using EMA to monitor rhinitis symptoms and using EMA for studies pertaining to low-income and racially minoritized populations, or other groups, with low technological literacy.

## Appendix

Multimedia Appendix 1 System Usability Scales.

## Abbreviations

AR: allergic rhinitis
EMA: ecological momentary assessment
ENT: Ear, Nose, and Throat
IAQ: indoor air quality
PM: particulate matter
PM2.5: particulate matter with a diameter of ≤2.5 µm
REDCap: Research Electronic Data Capture
SUS: System Usability Scale
TVOC: total volatile organic compound
VOC: volatile organic compound

## Data Availability

The datasets generated or analyzed during this study are available from the corresponding author upon reasonable request.
