# Supplementary material for: Assessing the Impact of Home Environmental Exposures on Allergic Rhinitis Using Real-Time Air Quality Monitoring and Symptom Assessment: Observational Feasibility Study
Source: JMIR Form Res. 2025 Jun 23;9:e73215. doi: 10.2196/73215 (PMC12235205; doi:10.2196/73215)
Supplement: Multimedia Appendix 1 [file formative_v9i1e73215_app1.docx]

| **The following questions are about your Awair air monitor.** | |
| --- | --- |
| Over the last two weeks, I used the Awair air monitor frequently. | O Strongly agree  O Somewhat agree  O Neither agree nor disagree  O Somewhat disagree  O Strongly disagree |
| I found the Awair monitor unnecessarily complex. | O Strongly agree  O Somewhat agree  O Neither agree nor disagree  O Somewhat disagree  O Strongly disagree |
| I thought the Awair air monitor. | O Strongly agree  O Somewhat agree  O Neither agree nor disagree  O Somewhat disagree  O Strongly disagree |
| I think that I would need the support of a technical person to be able to use the Awair air monitor. | O Strongly agree  O Somewhat agree  O Neither agree nor disagree  O Somewhat disagree  O Strongly disagree |
| I would imagine that most people would learn to use the Awair air monitor very quickly. | O Strongly agree  O Somewhat agree  O Neither agree nor disagree  O Somewhat disagree  O Strongly disagree |
| I found the Awair air monitor very cumbersome to use. | O Strongly agree  O Somewhat agree  O Neither agree nor disagree  O Somewhat disagree  O Strongly disagree |
| I felt confident using the Awair air monitor. | O Strongly agree  O Somewhat agree  O Neither agree nor disagree  O Somewhat disagree  O Strongly disagree |
| I needed to learn a lot of things before I could get going with the Awair air monitor. | O Strongly agree  O Somewhat agree  O Neither agree nor disagree  O Somewhat disagree  O Strongly disagree |
| I thought there was too much inconsistency with the Awair air monitor. | O Strongly agree  O Somewhat agree  O Neither agree nor disagree  O Somewhat disagree  O Strongly disagree |
| I found Awair air monitor were well integrated into the Home Air and Rhinitis Study. | O Strongly agree  O Somewhat agree  O Neither agree nor disagree  O Somewhat disagree  O Strongly disagree |
| What did you like/not like about the Awair air monitor in the study? |  |
| **The following questions are about your PiLR EMA Health app.** | |
| Over the last two weeks, I used the PiLR EMA Health app frequently. | O Strongly agree  O Somewhat agree  O Neither agree nor disagree  O Somewhat disagree  O Strongly disagree |
| I found the PiLR EMA Health app unnecessarily complex. | O Strongly agree  O Somewhat agree  O Neither agree nor disagree  O Somewhat disagree  O Strongly disagree |
| I thought the PiLR EMA Health app was easy to use. | O Strongly agree  O Somewhat agree  O Neither agree nor disagree  O Somewhat disagree  O Strongly disagree |
| I think that I would need the support of a technical person to be able to use the PiLR EMA Health app. | O Strongly agree  O Somewhat agree  O Neither agree nor disagree  O Somewhat disagree  O Strongly disagree |
| I would imagine that most people would learn to use the PiLR EMA Health app very quickly. | O Strongly agree  O Somewhat agree  O Neither agree nor disagree  O Somewhat disagree  O Strongly disagree |
| I found the PiLR EMA Health app very cumbersome to use. | O Strongly agree  O Somewhat agree  O Neither agree nor disagree  O Somewhat disagree  O Strongly disagree |
| I felt very confident using the PiLR EMA Health app. | O Strongly agree  O Somewhat agree  O Neither agree nor disagree  O Somewhat disagree  O Strongly disagree |
| I needed to learn a lot of things before I could get going with the PiLR EMA Health app. | O Strongly agree  O Somewhat agree  O Neither agree nor disagree  O Somewhat disagree  O Strongly disagree |
| I thought there was too much inconsistency with the PiLR EMA Health app. | O Strongly agree  O Somewhat agree  O Neither agree nor disagree  O Somewhat disagree  O Strongly disagree |
| I found the PiLR EMA Health app surveys to be well integrated into the Home Air and Rhinitis Study. | O Strongly agree  O Somewhat agree  O Neither agree nor disagree  O Somewhat disagree  O Strongly disagree |
| I found the PiLR EMA Health app surveys to be unnecessarily repetitive. | O Strongly agree  O Somewhat agree  O Neither agree nor disagree  O Somewhat disagree  O Strongly disagree |
| I found the PiLR EMA Health app surveys to be time consuming. | O Strongly agree  O Somewhat agree  O Neither agree nor disagree  O Somewhat disagree  O Strongly disagree |
| I found the PiLR EMA Health app surveys seems to arrive at an inconvenient time. | O Strongly agree  O Somewhat agree  O Neither agree nor disagree  O Somewhat disagree  O Strongly disagree |
| What did you like/not like about the PiLR EMA Health app in this study? |  |
| **The following are questions about the study as a whole.** | |
| I found the various components in the Home Air and Rhinitis Study were well integrated. | O Strongly agree  O Somewhat agree  O Neither agree nor disagree  O Somewhat disagree  O Strongly disagree |
| Is there anything else you would like to tell us about the Home Air and Rhinitis Study? |  |

System Usability Scale for Participants at University of Illinois Chicago

| Over the last two weeks, I used the Awair air monitor frequently. | O Strongly agree  O Somewhat agree  O Neither agree nor disagree  O Somewhat disagree  O Strongly disagree |
| --- | --- |
| I found the Awair monitor unnecessarily complex. | O Strongly agree  O Somewhat agree  O Neither agree nor disagree  O Somewhat disagree  O Strongly disagree |
| I thought the Awair air monitor was easy to use. | O Strongly agree  O Somewhat agree  O Neither agree nor disagree  O Somewhat disagree  O Strongly disagree |
| I think that I would need the support of a technical person to be able to use the Awair air monitor. | O Strongly agree  O Somewhat agree  O Neither agree nor disagree  O Somewhat disagree  O Strongly disagree |
| I would imagine that most people would learn to use the Awair air monitor very quickly. | O Strongly agree  O Somewhat agree  O Neither agree nor disagree  O Somewhat disagree  O Strongly disagree |
| I found the Awair air monitor very cumbersome to use. | O Strongly agree  O Somewhat agree  O Neither agree nor disagree  O Somewhat disagree  O Strongly disagree |
| I felt very confident using the Awair air monitor. | O Strongly agree  O Somewhat agree  O Neither agree nor disagree  O Somewhat disagree  O Strongly disagree |
| I needed to learn a lot of things before I could get going with the Awair air monitor. | O Strongly agree  O Somewhat agree  O Neither agree nor disagree  O Somewhat disagree  O Strongly disagree |
| I thought there was too much inconsistency with the Awair air monitor. | O Strongly agree  O Somewhat agree  O Neither agree nor disagree  O Somewhat disagree  O Strongly disagree |
| I found Awair air monitor were well integrated into the Home Air and Rhinitis Study. | O Strongly agree  O Somewhat agree  O Neither agree nor disagree  O Somewhat disagree  O Strongly disagree |
| What did you like/not like about the Awair air monitor in the study? |  |
